# RNAseq analysis reveals drought-responsive molecular pathways with candidate genes and putative molecular markers in root tissue of wheat

**DOI:** 10.1038/s41598-019-49915-2

**Published:** 2019-09-26

**Authors:** Mir Asif Iquebal, Pradeep Sharma, Rahul Singh Jasrotia, Sarika Jaiswal, Amandeep Kaur, Monika Saroha, U. B. Angadi, Sonia Sheoran, Rajender Singh, G. P. Singh, Anil Rai, Ratan Tiwari, Dinesh Kumar

**Affiliations:** 1 Centre for Agricultural Bioinformatics, ICAR-Indian Agricultural Statistics Research Institute, Library Avenue, PUSA, New Delhi, 110012 India; 2 ICAR-Indian Institute of Wheat and Barley Research, Karnal, Haryana 132001 India

**Keywords:** Biotechnology, Computational biology and bioinformatics

## Abstract

Drought is one of the major impediments in wheat productivity. Traditional breeding and marker assisted QTL introgression had limited success. Available wheat genomic and RNA-seq data can decipher novel drought tolerance mechanisms with putative candidate gene and marker discovery. Drought is first sensed by root tissue but limited information is available about how roots respond to drought stress. In this view, two contrasting genotypes, namely, NI5439 41 (drought tolerant) and WL711 (drought susceptible) were used to generate ~78.2 GB data for the responses of wheat roots to drought. A total of 45139 DEGs, 13820 TF, 288 miRNAs, 640 pathways and 435829 putative markers were obtained. Study reveals use of such data in QTL to QTN refinement by analysis on two model drought-responsive QTLs on chromosome 3B in wheat roots possessing 18 differentially regulated genes with 190 sequence variants (173 SNPs and 17 InDels). Gene regulatory networks showed 69 hub-genes integrating ABA dependent and independent pathways controlling sensing of drought, root growth, uptake regulation, purine metabolism, thiamine metabolism and antibiotics pathways, stomatal closure and senescence. Eleven SSR markers were validated in a panel of 18 diverse wheat varieties. For effective future use of findings, web genomic resources were developed. We report RNA-Seq approach on wheat roots describing the drought response mechanisms under field drought conditions along with genomic resources, warranted in endeavour of wheat productivity.

## Introduction

Bread wheat (*Triticum aestivum* L.) is the most widely grown crop of the world which is grown over 220 million hectares^1^. It caters staple food need of 30% of global population. Its water requirement is highest among all crops which is catered by rain and irrigation after maize and rice, respectively^1^. Inter-annual production variability of wheat is >40% due to heat waves and drought^2^. It has been projected that climate change may adversely affect the wheat production by 29%^3^. Since with one degree Celsius rise in global temperature, there is decline of wheat productivity by 6%, thus by 2080, projected global temperature of 4.5 degree Celsius will further widen the demand gap. Projected population of 10.8 billion by 2090 along with rise in global temperature and drought will reduce 9–10% annual yield, thus there is potential threat to food security in long run^2^ due to >60% demand rise which is going to be a global challenge^4^. To manage required wheat productivity, drought tolerant cultivars need to be developed to mitigate situation of famine and food crisis fetching economic and social stability^5^.

Since drought tolerance is a polygenic trait having genotype by environment interaction with low heritability, thus there is greater scope of varietal improvement by molecular breeding^6,7^. Transgenic approach for increase in drought tolerance has not contributed in development of drought tolerant wheat varieties^8^. Therefore, there is a greater need to accelerate conventional breeding program supplementing by associated molecular markers. Drought related quantitative trait loci (QTL)/gene identification for improvement of wheat varieties for drought tolerance has been reported^9^. Though QTL map of drought responsive traits under irrigated and rainfed environment are identified, but such analysis has limitation of poor resolution beyond 10cM^10^ and limited resolving power of the individual techniques^11^. QTL introgression may produce unexpected results^12^. Marker assisted selection (MAS) based on QTL linked markers has limited success in trait improvement^13^. This is due to very limited number of efficient QTL having major effect on phenotypic variation. Effect of minor QTL on phenotypic variability estimation are often biased, thus they are not that effective^14^. Discovery of QTL itself is not sufficient enough to incorporate them in breeding program as confidence interval (CI) of QTL in linkage analysis may span in multiples of ten map units thus it may have >100 genes. For successful use of QTL in selection program, it needs identification of specific polymorphism(s) which are having observed effect, thus QTL must be dissected into quantitative trait nucleotide (QTN) for more effective use^15^ by alternative approach for marker discovery using transcriptomic approach.

Drought adaptability mechanism in complex hexaploid genome needs interdisciplinary approach having water stress induced tissue specific phenotyping and its gene expression studies^16^. Wheat drought gene expression studies have been done by RNA Sequencing as well as proteomics approach^17,18^. Earlier studies/comparative studies of root microRNA and long non-coding RNAs of wild and bread wheat against drought response are reported^19,20^. Such comparative studies of miRNAs of bread wheat and its ancestors are also reported^21^.

Due to drastic reduction in costs of NGS, transcriptomic approach can contribute to decipher differential gene expression between contrasting varieties/genotypes along with SNP marker discovery especially by exposure of transcripts over available QTL map. Such approach can discover candidate genes controlling drought tolerance in wheat^22,23^. QTL can be further dissected at molecular level by using transcriptomic data to construct gene regulatory network (GRN) depicting key hub genes and regulatory mechanism associated with drought response^24^. Such data can be used to discover genic region derived markers for improvement of drought tolerance in wheat by gene pyramiding. Transcriptome sequencing has been used for marker development associated with drought tolerance, for example, in perennial grass, *Miscanthus* resulting into two markers for leaf relative water content and five markers for photosynthetic efficiency^25^. The transcriptomic approach has been used successfully in forage legume crop to obtain insight of drought response and associated candidate genes along with their markers^26^. Earlier transcriptomic studies of wheat drought stress were confined to leaves^27,28^, flower tissue and its different stages^29^. Roots have been proposed as a best choice of research to improve crop adaptation to drought stress conditions^30^. Roots are the first organs involved in drought sensing and exposure to water deficiency in soil. Root architecture plays an important role in drought adaptation. Though wheat drought transcriptomic studies are reported but have limitations like study on single genotype^31^, microarray based limited gene discovery^32,33^ and chemically induced drought in hydroponic system^34^. 

None of these are based on root tissues. Very recently, root based transcriptome study is reported^33,34^ but such studies does not cover root phenotyping based growth stage (Zadok scale) specific drought resilience mechanism where highest effect of drought stress on wheat productivity is known^35^. Earlier studies did not attempt to dissect known root drought QTL by mapping of the transcripts to decipher the genes and its expressional magnitude with marker discovery.

Contrasting genotypes differing in drought adaptive mechanisms may be used by transcriptomic approach to reveal associated signaling pathways which transfers signals towards root and shoot for molecular responses to fetch biochemical and morphological changes to protect water loss and tolerate stress^36^. Small and lateral root formation and change in its thickness act as an adaptive strategy to increase water uptake by providing more absorptive surface. Moreover, there has been report of QTLs controlling root growth angle under negative regulation of auxin playing important role in root drought response in crop^37^. Using transcriptome approach, associated candidate genes and its variant can further dissect such QTLs. In online web genomic resource, *WheatExp* database (https://wheat.pw.usda.gov/WheatExp/), there is no resource of drought transcriptome of root tissue having contrasting genotype. Data point and contrasting genotype are pivotal in transcriptomic investigation leading to discovery of candidate gene, associated pathways and genic region marker discovery. Root tissues for this study were selected at flag leaf stage. This stage is good for selection of better photosynthetic activity and yield^38^. In wheat breeding this has been used as morphological marker for QTL discovery as yield determinant^39^. In case of wheat, twenty stable QTLs for flag leaf morphology have been used for genetic improvement of drought tolerance^40^. It is well reported that this stage is having highest biomass growth rate due to highest photosynthetic activity which affects grain yield^41^. Beside these reasons, this stage is most sensitive growth stage for drought thus expected to have critical genetic mechanism of resilience which is practically relevant in breeding^42^. Also this stage offers advantage due to its strong positive correlation with other desirable traits of wheat productivity like spike length, kernel number, and weight per spike^43^. Extreme contrasting crop genotypes representing desert and Mediterranean climate has been successfully used to obtain significant differences in drought responsive mechanism in barley^44^. Drought responsive contrasting genotype of wheat has been reported to vary in its major mechanisms of nitrogen metabolism and carbon metabolism. In case of drought, such extreme genotype respond differentially for crop growth and yield by differential response of photosynthesis and nitrogen metabolism. Susceptible genotypes have been reported to show reduction in yield and yield stability unless they are in irrigated condition. Tolerant genotype has higher chlorophyll stability index along with higher membrane stability^45^. Thus root transcriptome of two contrasting wheat genotypes can be used to decipher drought responsive candidate genes and associated pathway.

Present investigation aims at identification of candidate genes in root at flag leaf booting stage in wheat using contrasting genotypes of drought tolerance and susceptibility by its transcriptional profiling along with gene regulatory network in response to water deprivation by irrigation withdrawal. Further, it aims to discover putative molecular markers (SSRs, SNPs and InDel markers), prediction of transcription factors (TFs) and microRNA binding sites with validation. It also aims to develop web genomic resources along with demonstration of model work dissecting known wheat root drought QTL for gene and marker discovery.

## Material and Methods

### Stress treatment, tissue collection and root phenotyping

Two contrasting genotypes of wheat, namely NI5439 (drought tolerant) and WL711 (drought susceptible) were used in this study. These were grown under well-watered and severe drought conditions. The two selected genotypes were sown in PVC pipe columns having 1.05 m length and 0.18 m diameter. Each pipe was filled with mixture of thoroughly mixed soil, sand and vermi-compost in 3:1:1 ratio, respectively. The well-watered treatment plants were kept at normal condition while the drought treated plants were placed in the transparent sheet covered area. Initially, three germinated seeds were sown in each pipe and later only one healthy seedling per pipe was retained at 15 days after sowing. The pipe were irrigated twice daily to maintain the soil moisture before the start of progressive soil drying. Drought stress was initiated at Z24 Zadok’s scale (main shoot and four tillers stage) and root tissues were taken at Z37 according to Zadok’s scale (flag-leaf just visible) and frozen immediately in liquid nitrogen and stored at −80 °C for further use^46^. Phenotypic data on root length, root diameter, root volume were also recorded. The intact soil along with roots were carefully removed from faces of the break part of the PVC pipes and further cut into 4 sections of 0–30 cm, 30–60 cm, 60–90 cm and 90–120cm^47^. The pipe was tapped so that the soil gets pulled out along with the whole plant. Roots were washed with slow pressure of water fountain on long (1.5 m) sieve to remove the soil, taking care not to damage the roots. Further samples were cut into 4 sections of 0–30 cm, 30–60 cm, 60–90 cm and 90–120 cm and kept in 70% alcohol. Then samples of each section were scanned on a document scanner and processed with WinRHIZO® software^48^.

### RNA Isolation and cDNA Library Construction

Total RNA was extracted from the root samples using Qiagen RNA isolation kit according to the manufacturer’s instructions. DNA was removed by digestion with RNase-free DNase and RNA was purified and concentrated using an RNeasy column (Qiagen, Germany). RNA quality was evaluated by 1% agarose gel electrophoresis for 28 S/18 S rRNA band intensity (2:1) and Agilent 2100 Bioanalyser. The samples were quantified using Nanodrop 2000 spectrophotometer (Thermo Fisher Scientific, USA).The A260/A280 nm ratios for all samples ranged between 1.8 and 2.1. Only the RNA samples with 260:280 ratio ranging between 1.9 and 2.1 and RNA integrity number (RIN) > 8.0 were used for further analysis. RNA-Seq libraries were prepared with Illumina TruSeq Stranded mRNA Sample Preparation Kit as per the manufacturer’s instructions. The experiment included two genotypes under two conditions (each data point pooled from ten plants), which resulted in four RNA-Seq libraries. Variation across samples were minimised by pooled root tissues from 10 different plants in each set of control and treatment^49^. These libraries were sequenced on Illumina HiSeq 2000 platform (Illumina, San Diego, CA) with 100 nucleotide pair-end reads. The libraries were labelled as Tolerant Control (TC), Tolerant Drought (TD), Susceptible Control (SC) and Susceptible Drought (SD) and submitted to the SRA of NCBI having BioProject: PRJNA432496 (BioSamples: SAMN08450194, SAMN08450195, SAMN08450196, SAMN08450197).

### Pre-processing and *de novo* assembly

Quality assessment of control and stressed wheat cultivars viz. NI5439 (tolerant) and WL711 (susceptible) was performed using FASTQC tool^50^. Pre-processing and removal of low quality reads (phred-like q value ≤ 20), adapters was carried out using Trimmomatic tool version 0.33^51^. Further, high quality filtered reads of all the four samples were pooled together and *de novo* wheat transcriptome assembly was done using Trinity v2.0.6 assembler. For assembly of short reads, Trinity uses de Bruijn graph algorithm and default k-mer value i.e. 25^52^. Finally, CAP3 assembler was run on Trinity generated assembly for the removal of redundant sequences^53^. In root transcriptome analysis, fungal transcripts are usually present which were removed for analysis^54^. The sequences showing the BLAST hits with contaminating fungal sequences were removed from the datasets for further analysis.

### Differential Gene Expression Analysis

To obtain the read density and gap free alignment, paired-end reads of four samples were separately mapped onto *de novo* wheat transcriptome assembly using Bowtie^55^. RNA-Seq by Expectation-Maximization (RSEM) tool was used to calculate abundance estimation and expression value of each transcript^56^. Further, differentially expressed genes (DEGs) were identified using edgeR package^57^. For identification of significant genes, stringent parameters such as log2FC ± 5 and false discovery rate (FDR) < 0.001 were applied. Two different tools, namely, edgeR and NOISeq were used to reduce the noise with better computational reproducibility. NOISeq gives better result with unreplicated data due to its non-parametric and data-adaptive approach while computing the DEGs^58^. To identify significant DEG, threshold variance with (p-value 0.05) was set^59,60^. The results of both the tools were compared at 0.99 q value to establish reliability of findings by edgeR.

### Validation of DEGs through RT-qPCR

To validate the RNA-seq results, 12 DEGs were randomly selected (Supplementary Table S5). The cDNA was prepared through Superscript® III First Strand Synthesis System (Invitrogen, UK) for qRT-PCR as per manufacturer’s instructions. Before proceeding to qPCR, the optimization for corresponding target genes were performed by using routine PCR. After PCR confirmation, qPCR was done in a reaction volume of 10 μl containing10ng/μl of cDNA, 5 μl of 2 × SYBR Green Master Mix (Thermo Scientific) and 1 μl each of forward and reverse primer. The quantitative reaction was done on Bio-Rad CFX96™ Real-Time PCR System (Bio-Rad, USA). The qPCR program consisted of 95 °C for 5 min, then 40 cycles of 94 °C for 15 s, 58 °C for 30 s and 72 °C for 30 s and a final melt curve step from 65° to 95 °C with a rise of 0.5 °C for 5 s. The gene expression values were normalized against an internal reference gene, actin. The reactions were performed in three biological replicates. The relative expression level of selected transcripts were normalized with actin by comparative 2−ΔΔCt method.

### Annotation and functional characterization of DEGs

Standalone BLASTX program was used to find putative function of differentially expressed genes against NCBI non-redundant database (ftp://ftp.ncbi.nlm.nih.gov/blast/db/) with threshold e value 0.05^61^. Analysis of Gene ontology (GO) and KEGG (Kyoto Encyclopedia of Genes and Genomes) pathways was performed using Blast2Go Pro version 3.1^62^. Gene ontology (GO) term analysis categorized transcripts into three major functional groups namely, molecular functions, biological processes and cellular components.

### Prediction of TF and miRNA

Blastx search (e-value 1e-05) was used for identification of transcriptional factors (TFs) against PlantTFDB (Plant Transcriptional Factors Database) version 4.0^63^ for each of the four sets, namely, TC:TD, SD:TD, SC:SD and SC:TC. Further, 119 mature miRNA of *Triticum aestivum* were used for predicting their targets in the differentially expressed genes using psRNATarget webserver^64,65^ (http://www.mirbase.org/). For validation of predicted miRNA from each set, their BLAST analysis with strict stringency criteria such as 100% identity and zero mismatch against publically available wheat root drought specific small RNA library (NCBI/SRA SRR1055298) was done.

### Gene regulatory network analysis

Cytoscape (version 3.2.1)^66^ tool was used for analysis of gene network analysis of differential expressed genes. For network analysis, top 100 upregulated and downregulated genes each were considered. ARACNE (Algorithm for the Reconstruction of Accurate Cellular Networks) and Network Analyzer plug-in were used for analysing the network of all the four sets of DEGs. On the basis of high degree and betweenness, hub genes were selected.

### Discovery of markers

The putative Simple Sequence Repeats (SSRs) and variants were predicted from *de novo* transcriptome assembly of wheat. SSR Markers were predicted using perl scripts of MISA (MIcroSAtellite identification tool)^67^. For mining of significant markers, ten repeating units for mononucleotides, six repeating units for dinucleotides and five repeating units for trinucleotides, tetranucleotides, pentanucleotides and hexanucleotides were taken. Primers were generated using PRIMER3 tool^68^.

To find the variants (SNPs and InDels), we used two references, i.e., our constructed wheat *de novo* transcriptome assembly and wheat genome release version 31 (ftp://ftp.ensemblgenomes.org/pub/plants/release-31/fasta/triticum_aestivum/dna/). All the transcripts were mapped using Burrows-Wheeler Aligner (BWA) tool^69^. SAM tools package was used for calling SNPs and Indels^70^. For obtaining of significant variants, several stringent parameters were used such as read depth ≥15^71^ and quality >30^72,73^. To visualize the relative distribution of SNPs over 21 chromosomes, Circos tool was used^74^.

### Validation of SSR markers

Fifteen SSR markers randomly chosen were used for validation in a panel of 18 wheat genotypes, selected from the mini-core set developed for the drought tolerance studies (Supplementary Table S11). Genomic DNA was extracted from seedlings by CTAB method. DNA amplification was carried out in a 25 μL reaction mixture containing 2.5 μL 10 × buffer,0.5 μL of 10 mM dNTPs, 0.5 μL of 10 μM each reverse and forward primer, 0.125 μL of Taq polymerase, and 60 ng template DNA. PCR amplification was performed on BioRad S1000™ using the program: 94 °C for 4 min, 30 cycles of 1 min at 94 °C, 50 s at 55–63 °C, 72 °C for 1 min and a final cycle of 7 min at 72 °C. The primers that were not successful for amplification were reanalyzed using gradient PCR method. Electrophoresis was performed on 3% low EEO gel.

### Mapping of DEG transcripts over chromosome 3B root drought QTL region

Similarity search of *de novo* assembly as well as DEGs was performed using Blastn against *Triticum aestivum* whole genome release 31 (ftp://ftp.ensemblgenomes.org/pub/plants/release-31/fasta/triticum_aestivum/dna/). In a study of drought responsive nine traits and associated QTL discovery in wheat, maximum QTLs were found to be present on chromosomes 3B and 4A^10^. Out of these two chromosomes, major QTL affecting root drought response has been reported on chromosome 3^75^. In order to visualize root expressional QTL, chromosome 3B was selected for mapping of transcripts. Two well-known QTLs of wheat root trait in response to drought (Xbarc268- Xbarc075 and Xbarc102-Xbarc268) were taken from literature^76^. Primer sequence of these QTL markers were also obtained from WheatIS (wheat information system) of INRA (http://www.wheatis.org/index.php) were used for ePCR over wheat chromosome 3B to locate the starting position of these two QTLs on wheat genetic map. Since it is the relative distance, it was converted into basepair for physical mapping to show its location in physical map. Since cM distance varies from species to species and also varies from chromosome to chromosome in a given species, thus it requires specific conversion factor. For wheat chromosome 3B, the specific conversion factor 0.7 was used^77^. The genes in these mapped regions were further subjected to SNP discovery in transcripts to enlist the QTL regions harboring potential QTNs.

### Web genomic resources

An online relational database of wheat drought transcriptome was developed which catalogues differentially expressed genes, miRNAs, transcription factors, KEGG pathways along with markers (SSRs, SNPs and InDels). This web-resource is based on “three-tier architecture” having, client-, middle- and database tier. This genomic resource can be accessed freely for non-commercial use at http://webtom.cabgrid.res.in/wdrotdb/. The client tier is concerned with browsing and user query through web pages. MySQL in the database tier stores all the information related to DEGs, TFs, KEGG pathways and markers in tabular form. For database connectivity, execution and fetching of query, server side scripting was done in PHP in the middle tier.

## Results and Discussion

### Root phenotyping for the differential drought tolerance of wheat genotypes

Root phenotyping for drought tolerance was done successfully for both the sets, namely, control and treated. It was observed that in set where drought was induced by water stress, the root length (in section 90–120 cm) and average diameter (30–60 cm) of susceptible variety WL711 were reduced significantly at 5% as compared to control without having water stress. In drought-tolerant variety NI5439, root length, total surface area and length/volume increased significantly (p value = 0.05) upto 30–60 cm depth by drought treatment as compared to control (Supplementary Table S1).

### Transcriptome data generation

RNA was isolated successfully from both the sets with RIN value > 8.0. RNA-Seq libraries were made successfully to generate transcriptomic data using Illumina HiSeq. 2000 platform. A total of 78.2 GB data (paired end of 100*2) were generated.

### Pre-processing and *de novo* assembly

After pre-processing of reads, total 1073961 low quality reads from stress and control samples of namely NI5439 (tolerant) and WL711 (susceptible) genotypes were removed. Finally, 161971774 high quality reads of all the samples were pooled together for *de novo* transcriptome assembly. Trinity assembler generated a total of 370488 transcripts with N50 value 1106 bp. Further, CAP3 assembler tool was employed on 370488 Trinity assembled transcripts for the removal of redundant sequences. Finally 365752 transcripts were obtained by CAP3 with N50 value of 1092 bp and GC content 49.46%. In assembly results, minimum and maximum sequence lengths were 301 and 29228 bp, respectively (Table 1).

**Table 1:** Summary statistics of assembly.

Total number of sequences	365752
Total length of sequence	306828579 bp
GC %	49.46%
Total GC count	151762458 bp
N25 stats	>=1927 bp
N50 stats	>=1092 bp
N75 stats	>=586 bp

### Differential gene expression analysis

A total of 45139 and 44919 DEGs were obtained from the four sets (Tolerant Control vs. Tolerant Drought - (TC:TD), Susceptible Drought vs. Tolerant Drought - (SD:TD), Susceptible Control vs. Susceptible Drought - (SC:SD), Susceptible Control vs. Tolerant Control - (SC:TC)) in comparison, using edgeR and NOISeq methods, respectively. Comparative analysis of both methods were performed in all the four sets. More than 83% were common except SC:SD (77%). Further, comparison of top 200 from each of up and down regulated differentially expressed transcripts >80% were found to be in common. Interestingly, two (SD:TD and SC:TC) of these were >95% common (Table 2) reflecting reliability of findings by edgeR. Paired-end reads of control and drought stressed samples of both contrasting varieties were separately mapped onto *de novo* transcriptome assembly for abundance estimation of transcripts in the form of FPKM (fragments mapped per kilo base of exon per million reads mapped). Since transcriptome data has been generated from root tissue which was in direct contact with soil, thus it is obvious to find microbial unigenes. Six different fungal genera namely, *Alternaria alternata*, *Ascochyta rabiei*, *Fusarium* spp., *Hypsizygus marmoreus*, *Plasmodiophora brassicae* and *Phytophthora* spp. were observed which were removed from all the four sets. Finally 17798, 8103, 9910 and 9328 differential expressed genes were observed in the sets TC:TD, SD:TD, SC:SD and SC:TC, respectively. Out of these differentially expressed genes, a total of 12093, 4245, 4915 and 2486 genes were up-regulated in the sets TC:TD, SD:TD, SC:SD and SC:TC, respectively (Table 3, Supplementary Table S2). These sets are having higher number (45139) of transcripts than the earlier report^33^ having 8197 transcripts. These higher number of transcripts obtained in this study could be used for genomic resource development also.

**Table 2:** Comparison of differentially expressed transcripts by edgeR and NOISeq.

S. No.	Data sets	edgeR	NOIseq	Common total transcripts obtained	Common top 200 up and down regulated transcripts
		total (FC = 5 and FDR = 0.001)	Q = 0.99		
1	TC:TD	17798	17339	15139 (85%)	319 (80%)
2	SD:TD	8103	8603	6782 (83%)	391 (98%)
3	SC:SD	9910	9370	7683 (77%)	325 (81%)
4	SC:TC	9328	9607	7756 (83%)	378 (95%)

**Table 3:** Upregulated and downregulated differential expressed genes in four sets viz., TC:TD, SD:TD, SC:SD and SC:TC.

S. No.	Data sets	Total (FC = 2 and FDR = 0.05)	After removal of Fungal and Oomycete transcripts	Total (FC = 5 and FDR = 0.001)
1	TC:TD	51275	36866	17798 Up-12093 Down-5705
2	SD:TD	19098	14819	8103 Up-4245 Down-3858
3	SC:SD	32,472	22225	9910 Up-4915 Down-4995
4	SC:TC	39173	28026	9328 Up-2486 Down-6842

### Annotation and functional characterization of DEGs

**Figure 1:**
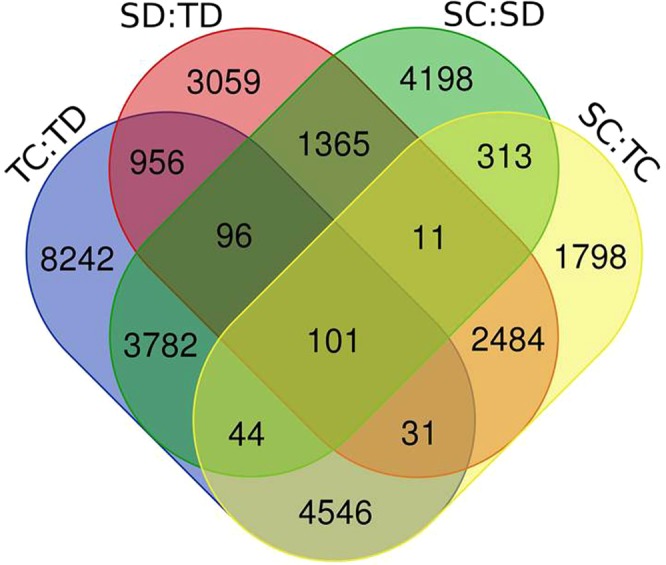
Venn diagram showing shared and unique DEGs of wheat root transcriptome associated with drought.

Annotation of four sets namely, TC:TD, SD:TD, SC:SD and SC:TC revealed maximum similarity with its wild species progenitor, *Aegilops tauschii* followed by red wild einkorn wheat (*Triticum urartu*) and barley (*Hordeum vulgare*) (Supplementary Table S2) which is due to their phylogenetic similarity. KEGG pathway analysis revealed purine metabolism, thiamine metabolism and biosynthesis of antibiotics pathways in all the four sets with different numbers of transcripts involved (Supplementary Table S3). Annotation similarity among these species also reflects that drought response core pathways and mechanism are well conserved in these species with few species specific variation. We found 6175 unique transcripts without any hit in the annotation analysis. Potential reason of this could be: 1) novel transcripts specific to species bread wheat, 2) Alignment error in transcriptome assembly due to shorter reads of Illumina^78^, 3) Alignment error due to gene families of A, B and D genomes^79^. In order to depict the distribution of putative candidate genes of wheat drought response, Venn diagram was constructed comparing the four sets of DEGs. A set of 101 genes were found common to all (Fig. 1), while 8242, 3059, 4198 and 1789 DEGs were found unique in TC:TD, SD:TD, SC:SD and SC:TC, respectively. Fold change, FDR values and gene description of all the four sets are provided in Supplementary Table S4.

### Experimental validation of differential expression data by qRT-PCR

**Figure 2:**
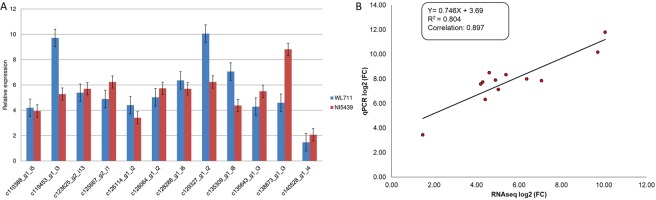
(**A**) Quantitative real-time PCR analysis of selected transcripts; (**B**) Correlation between magnitude of gene expression by FPKM and qPCR method.

Magnitude of expression of DEGs was validated by qRT-PCR analysis. For this, a total of 12 transcripts were selected randomly. Log fold change values of these selected transcripts were found largely in correspondence with qRT-PCR results (Fig. 2, Supplementary Table S5).

### Gene regulatory network and molecular mediation of wheat root drought response

There are two major pathways for drought response mediation in crops namely, ABA-dependent and ABA-independent which is also known as DREB-(dehydration-responsive element binding protein-) mediated pathway^9^. In our dataset, we found the key genes involved in both the pathways. We observed differential expression of signalling machinery regulating physiological response of drought in enlisted DEGs. For example, MAPK genes which are reported to be highly expressed in response to various abiotic stresses. In rice, overexpression of the MAP kinase is reported to be associated with drought tolerance^80^.

Among the differentially expressed genes, some are well known to mediate drought response by respective transcription factor based regulation, for example auxin response^81^, WRKY transcription factor^82^, HSF family mediating MPK3/MPK6 signaling^83^, AP2-like ethylene-responsive transcription factor^84^, MYB transcription factor^9^ and NAC transcription factor^85^. Similarly, genes well known for drought responsive pathways were found differentially expressed like Ran-binding proteins^86^, Peroxidase^87^, Lipoxygenase^88^ and LRR receptor-like serine/threonine-protein^89^.

**Figure 3:**
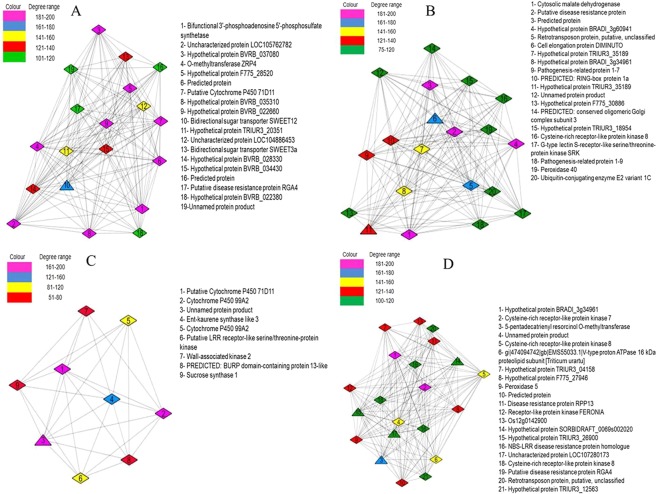
Gene regulatory network of wheat root transcriptome associated with drought. Figures A, B, C and D represents the network (TC:TD), (SD:TD), (SC:SD), and (SC:TC), respectively.

DEGs can be used for construction of GRN^90^. Logical model of GRN can be constructed with limited sample size to understand the co-expressional network and cross talk between key genes associated with the trait^91^. SNPs of genes involved in such GRN have been found to be regulating phenotype or trait^92^. The top 100 up and down regulated DEGs were used to construct GRN for each of the four sets. Based on the parameters namely degree and betweenness centrality 19, 20, 9 and 21 hub genes from (TC:TD), (SD:TD), (SC:SD), and (SC:TC) were found, respectively. In our results, we found maximum hub genes to be upregulated (Fig. 3, Supplementary Table S6).

Breeding drought tolerant crop has major challenge as there are several overlapping traits with complex network and cross talk between network genes^93^. Hub genes can be used further in deciphering of QTL network affecting multiple trait, multi QTL networking and association. More than 100 heat and drought adaptive QTL of wheat are identified in which some are overlapping. For example, one yield trait QTL located on 4A also accounts for 27 and 17% variation under drought and heat stress, respectively^94^. Hub genes have been reported to exhibit pleiotropic effect which may be tissue and time specific^95^.

We found major transcriptional differences in response to drought treatment by two contrasting wheat varieties in their comparative analysis (SD:TD). In our GRN (Fig. 3B) at least five hub genes having upregulation are well known to mediate drought tolerance. Our computed GRN of SD:TD (Fig. 3B) clearly depicts the major cardinal differences among two different genetic background of contrasting varieties. Here, sensing of drought by upregulated G-type lectin S-receptor-like serine/threonine-protein kinase SRK can be seen^96^. Similarly, upregulated disease resistance proteins of NBS-LRR and RPP13 induce salicylic acid pathway^97^. Antioxidative stress response is mediated by cytosolic malate dehydrogenase (CMD) as reported in *Arabidopsis*^98^. Cysteine-rich receptor-like protein kinase 8 is also known to control seedling growth arrest and stomatal closure in response to drought^99^.

Apart from hub genes, drought tolerant variety was observed with upregulation of at least seven well known genes reported to be associated with improved tolerance in various crops. Secondary metabolite production is known to provide better drought tolerance in plants^100^. We found upregulation of amidase and lovastatin nonaketide synthase-like protein genes which are known for similar secondary metabolite production. These various cellular processes are required for survival against osmotic and oxidative stress generated by drought^101,102^. Drought stress leads to increase in level of H_2_O_2_ in root which is controlled by upregulation of peroxidase as reported in other drought tolerant wheat varieties^103^. Upregulated RPM1 and family of stress-induced proteins are known to modulate abiotic stress including drought in *Arabidopsis*^104^ and bentgrass^105^, respectively. Family of PR proteins which are found upregulated in this comparative set are also well reported to provide biotic and abiotic stress tolerance in various crops^106^. Upregulated subtilisin-like serine protease gene which is also called SDD1 is reported to be associated with tolerance of drought, maintaining water balance at physiological level. This gene also controls stomatal density and guard cell formation offering further improved tolerance at developmental level of plant^107^.

In set of SD:TD, we found downregulation of hub gene family cytochrome P450 which is reported to have precise balance mechanism between its biosynthesis and catabolism controlled by ABA level in plants^97^. Among the other downregulated genes like Arginine Decarboxylase (ADC) is reported to control lateral root growth against drought in *Arabidopsis*^108^. Water retention in tolerant varieties is facilitated by lower expression of vacuolar-processing enzyme^109^. Pooling of soluble carbohydrate is required to maintain better osmotic balance for tolerance. For such activity, mediated by lower expression of fructan 6-fructosyltransferase is also reported in tobacco^110^. Potassium ion transport into apoplast and mediation of signal transduction from root to shoot via xylem sap is integral part of drought response by downregulation of alcohol dehydrogenase, which is also observed in our comparative set^111^.

Present finding describes drought response by *in situ* approach of root RNASeq analysis unlike previous reported hydroponic studies. We found drought sensing and transport control mechanism by root tissue in its gene regulatory network. Our study clearly reveals the mechanism of drought response by root tissues of wheat in soil, right from sensing of water deficiency by root, sensing of sucrose accumulation, intracellular signal transduction mediated by G-type lectin S-receptor-like serine/threonine-protein kinase SRK, ABA signalling along with salicylic acid pathway activation, oxidative stress response and ROS scavenging, energy balance, stomatal closure, regulation of cell wall, defense response and finally to the senescence (Table 4). This study of wheat drought induction in field by irrigation withdrawal in a natural system at Zadok’s scale Z24-Z37 is critical for drought resilience mechanism. It can supplement the earlier finding of Hu *et al*., 2018 which was based on chemically induced (PEG) drought in artificial hydroponic system^33^.

**Table 4:** Role of hub genes in all the four sets: TC:TD, SD:TD, SC:SD and SC:TC.

SD:TD	Description	Reference
Cytosolic Malate Dehydrogenase	It has been reported in *Arabidopsis* that it mediates protection against oxidative stress during drought to increase tolerance	^98^
Disease resistance proteins	Disease resistance proteins induce salicylic acid pathway for physiological adaptation against abiotic stress for improved tolerance	^97^
Retrotransposon protein	This gene is reported to be involved in ABA signalling pathway along with MPK6 in wild wheat relative to provide drought endurance	^120^
Cell elongation protein	This gene is reported to downregulate cell elongation in response to severe water deficiency interrupting water flow from xylem to surrounding elongated cells	^133^
Pathogenesis Related (PR) protein	This gene mediates role in sensing the water deficiency in root and regulates stress signaling and regulatory network controlling its targeted genes in the network	^36^
Ring Box	This gene codes for proteins having five common characteristic motifs, namely, RING domain, trans-membrane domain, basic amino-acids rich region, conserved GLD tripeptide. It is reported to be negative regulator of cold stress and positive regulator of drought stress in *Arabidopsis*	^134^
Conserved Oligomeric Golgi complex subunit (COG)	This gene mediates resistance against environmental stress by controlling cell wall growth regulation and defense response	^135, 136^
Cysteine-rich receptor-like protein kinase 8 [*Triticum urartu*]	It increases ABA sensitivity controlling seedling growth arrest and stomatal closure	^99^
G-type lectin S-receptor-like serine/threonine-protein kinase SRK [*Triticum urartu*]	Highly conserved, vital role in sensing outside signal of abiotic stress including drought, also known to control chlorophyll content, ion transport and plant height.	^96^
pathogenesis-related protein 1–9 [*Triticum aestivum*]	Apart from pathogen attack, PR-10 proteins were also induced by abiotic stresses like salinity, drought, copper, oxidative stress, or ultraviolet (UV) radiation.	^137^
Peroxidase 40 [*Aegilops tauschii*]	Its higher expression is reported in drought tolerant wheat varieties. Since ROS concentration is increased in drought thus to avoid cellular damage detoxification is done by this gene.	^138^
Ubiquitin-conjugating enzyme E2 variant 1 C [*Aegilops tauschii*]	The ubiquitin–proteasome system acts as central modifier of plant signaling in targeted protein degradation during drought induced senescence.	^139^
**TC:TD**		
Bifunctional 3’-phosphoadenosine 5’-phosphosulfate synthetase [*Aegilops tauschii*]	It is reported to be a multifaceted modulator of drought and high-light signalling in *Arabidopsis*	^140^
O-methyltransferase ZRP4 [*Aegilops tauschii*]	Controls synthesis of lignin, ferulate and wall phenolics controlling mechanical strength of cell walls in drought.	^141^
putative Cytochrome P450 71D11 [*Aegilops tauschii*]	It controls the level of ABA in plants by precise balance mechanism between its biosynthesis and catabolism.	^142^
Bidirectional sugar transporter SWEET12 [*Triticum urartu*]	sucrose accumulation is observed in plants exposed to low temperatures, drought and salt stress, and nutrient deficiency. Sucrose is sensed by the plant	^143^
Putative disease resistance protein RGA4 [*Aegilops tauschii*]	Disease resistance proteins induce salicylic acid pathway for physiological adaptation against abiotic stress for improved tolerance	^97^
**SC:SD**		
putative Cytochrome P450 71D11 [*Aegilops tauschii*]	It controls the level of ABA in plants by precise balance mechanism between its biosynthesis and catabolism.	^142^
Cytochrome P450 99A2 [*Aegilops tauschii*]	It controls the level of ABA in plants by precise balance mechanism between its biosynthesis and catabolism.	^142^
ent-kaurene synthase like 3 [*Triticum aestivum*]	Mediates transcriptional regulatory network and signaling regulation crop growth response against abiotic stress	^143^
Cytochrome P450 99A2 [*Triticum urartu*]	It controls the level of ABA in plants by precise balance mechanism between its biosynthesis and catabolism.	^142^
Putative LRR receptor-like serine/threonine-protein kinase [*Aegilops tauschii*]	Reported to control stomatal density in the leaf epidermis of rice in response to salt and drought stresses.	^89^
wall-associated kinase 2 [*Triticum aestivum*]	Transmembrane protein which perceives stimuli by their extracellular domains and transmits the signals via their cytoplasmic kinase domains in response to abiotic stress controlling cell elongation and development of root.	^144^
PREDICTED: BURP domain-containing protein 13-like [*Setaria italica*]	It is up regulated by salt, ABA and osmotic stress and down regulated by salicylic acid playing role in adaptation of stresses.	^145^
Sucrose synthase 1 [*Aegilops tauschii*]	Controls sucrose synthesis/metabolism in non-photosynthetic tissues, acts as osmoticum-sensing pathway via ABA-independent sensing. Also involved in phloem loading/unloading in response to drought.	^146^
**SC:TC**		
Cysteine-rich receptor-like protein kinase 7 [*Triticum urartu*]	It increases ABA sensitivity controlling seedling growth arrest and stomatal closure	^99^
5-pentadecatrienyl resorcinol O-methyltransferase [*Triticum urartu*]	Controls synthesis of lignin, ferulate and wall phenolics controlling mechanical strength of cell walls in drought.	^141^
Cysteine-rich receptor-like protein kinase 8 [*Triticum urartu*]	It increases ABA sensitivity controlling seedling growth arrest and stomatal closure	^99^
V-type proton ATPase 16 kDa proteolipid subunit [*Triticum urartu*]	Controls electrochemical proton gradient across tonoplast with sodium sequestration in vacuole enhancing abiotic stress tolerance in wheat.	^147^
Peroxidase 5 [*Aegilops tauschii*]	Its higher expression is reported in drought tolerant wheat varieties. Since ROS concentration is increased in drought thus to avoid cellular damage detoxification is done by this gene.	^138^
Disease resistance protein RPP13 [*Aegilops tauschii*]	Disease resistance proteins induce salicylic acid pathway for physiological adaptation against abiotic stress for improved tolerance	^97^
Receptor-like protein kinase FERONIA [*Aegilops tauschii*]	Mediates ABA activation of FER along with cross-talk between ABA and peptide hormone RALF controlling plant growth against stress stimuli.	^148^
NBS-LRR disease resistance protein homologue [*Hordeum vulgare*]	Disease resistance proteins induce salicylic acid pathway for physiological adaptation against abiotic stress for improved tolerance	^97^
Cysteine-rich receptor-like protein kinase 8 [*Triticum urartu*]	It increases ABA sensitivity controlling seedling growth arrest and stomatal closure	^99^
Putative disease resistance protein RGA4 [*Aegilops tauschii*]	Disease resistance proteins induce salicylic acid pathway for physiological adaptation against abiotic stress for improved tolerance	^97^
retrotransposon protein, putative, unclassified, expressed [*Oryza sativa Japonica* Group]	This gene is reported to be involved in ABA signalling pathway along with MPK6 in wild wheat relative to provide drought endurance	^120^

### Prediction of TF and miRNA

TF were predicted for each of the four sets, namely. TC:TD, SD:TD, SC:SD and SC:TC using BLAST search against PlantTFDB (Plant Transcriptional Factor Database). A total of 4722, 3093, 2739 and 3266 TF were found in TC:TD, SD:TD, SC:SD and SC:TC sets, respectively. In set TC:TD, maximum transcripts matched with bHLH (537), MYB (451) and NAC (328). These TFs have been extensively observed earlier in many plants under several different stress conditions mediating ABA dependent pathways^112^. In the set SD:TD, WRKY (327), FAR1 (256) and MYB (243) were more abundant (Supplementary Table S7). FAR1 leads to constitutive deactivation of cell death and decreasing the accumulation of reactive oxygen species (ROS), developed under stress^113^. MYB and bHLH factors are known to regulate root hair development and vacuolar acidification^114^. In SC:SD, bHLH (276), MYB (250) and NAC (182) were most abundant. NAC gene is reported to play important role in improvement of root growth imparting drought tolerance in cotton and *Arabidopsis*^115^. In the set SC:TC, WRKY (340), MYB (293) and bHLH (280) transcriptional factors were most abundant which are well known TFs in drought tolerance in crops (Supplementary Table S7).

In order to enlist putative microRNA, transcripts were used in miRBase which predicts on the basis of specific binding sites. Prediction was done for each of the four sets, namely TC:TD, SD:TD, SC:SD and SC:TC. In set TC:TD, 60 transcripts were identified which were targeted by 26 wheat miRNAs. Maximum abundance in our results were for miRNAs tae-miR1130b-3p, tae-miR1128 and tae-miR1133 having binding sites for 9, 8 and 7 transcripts, respectively. In set SD:TD, 83 transcripts targeted for 35 wheat miRNAs having maximum abundance of tae-miR1130b-3p, tae-miR1128 and tae-miR1133 against 12, 11 and 11 transcripts, respectively. It is interesting to note that drought associated miRNA, miR1130b is highly conserved and has been found in tetraploid wild wheat and hexaploid modern wheat^19,20^. In the set SC:SD, 24 miRNAs were targeting 56 transcripts with abundance of tae-miR1130b-3p, tae-miR5049–3p and tae-miR1128 of 11, 5 and 5 transcripts, respectively. Such high abundance of drought associated miR1130b and miR5049 is reported in several studies^16,20,21,116^.

For the set SC:TC, 36 miRNAs were found targeting 62 transcripts with maximum abundance of tae-miR1130b-3p, tae-miR1128 and tae-miR5049-3p in 10, 7 and 6 transcripts, respectively (Supplementary Table S8). Interestingly, BLAST analysis of predicted miRNAs against wheat root drought specific small RNA library revealed >80% of them present in each of the four sets (Table 5). Some of these enlisted miRNAs are reported for their role in mediating drought response in wheat, for example, miR1120c-5p, miR1127b-3p, miR5384-3p^117^ and miR5049, miR164, miR5048, miR159^118^, hence proving the validation of predicted miRNA by computational methods.

**Table 5:** Wheat drought responsive predicted miRNA and their validation.

Experimental sets	Number of predicted miRNA having target on DEGs	Number of miRNAs detected in small RNA library of wheat root tissue	Percentage of miRNAs validated in small RNA library of wheat root tissue
T(C)T(DS)	67	55	82.09
S(DS)T(DS)	95	82	86.32
S(C)S(DS)	56	48	85.71
S(C)T(C)	70	61	87.14

Our enlisted miRNA available on web genomic resources can be used for targeted discovery of SNPs to discover miRNA polymorphism which can be used in trait association studies. Since SNPs can affect secondary structure of stem regions and mature miRNAs target interactions thus, they can affect biogenesis and putative functions related to the trait^119^. Such miRNA polymorphism has already been reported controlling plant traits, for example, in case of rice miRNA osa-smR5864w having C/G point mutation is associated with pollen fertility/sterility. In case of wheat, point mutation A/G on the binding site of gene *TaMYB2* is associated with dehydration tolerance across varieties^119^.

As abiotic stress regulation is mediated by miRNAs and TFs, thus these findings are not only helpful in knowledge enrichment regarding drought response regulation but can also be used as genomic resource for drought improvement in wheat^116,120^. Enlisted miRNAs can be used for further functional characterization for crop improvement program^121^.

### Mapping of differentially expressed transcripts on chromosome 3B-localized drought-responsive root QTL

We have done BLAST of *de novo* transcriptome assembly and DEGs against wheat genome. Out of 365752 transcripts, 229729 transcripts were matched with wheat genome chromosome with greater than 70% identity and threshold e-value 0.001. In case of DEGs, we took 31418 DEGs after removal of duplicate and found 13171 DEGs which showed similarity with wheat genome. In both cases maximum transcripts were matched with chromosome 3B i.e. 23378 in *de novo* and 1430 in DEGs, followed by chromosome 2B i.e. 14998 and 958 in *de novo* and DEGs, respectively which may be due to its large size (data available at download section http://webtom.cabgrid.res.in/wdrotdb/).

A total of 88 QTLs are reported to explain 3.33–77.01% variability of root drought responsiveness in wheat seedling. These QTLs are present on chromosomes 1A, 1B, 1D, 2A, 2B, 2D, 3A, 3B, 4A, 4B, 4D, 5A, 5B, 5D, 6A, 6B, 6D, 7A, 7B and 7D^72^. This study reports many limitations like variation in QTL discovery between conditional and unconditional analysis as QTLs with effects lower than a certain threshold becomes virtually undetectable. In this interesting study, two QTLs have been detected between *Xbarc102-Xbarc268* and *Xbarc268-Xbarc075* on chromosome 3B. The first QTL is reported to have negative additive effect on root to shoot dry weight ratio (RSDWR) under osmotic stress whereas second QTL is known to have pleiotropic effects on many root traits like root fresh weight, length, number etc^76^.

Since structural variation in terms of SNP and Indel over these QTL regions has not been dissected further^76^, thus such studies have limitation of resolution up to gene level. This limitation is a major impediment in effective utilization of QTL as marker in association studies for molecular breeding program. For example, a wheat drought QTL present on chromosome 6A cannot be effectively utilized as they cannot be refined due to poor recombination frequency which is further compounded by paucity of SSR markers at the terminal location. To resolve such situation, transcriptome based studies can be used to discover candidate genes along with genic region SSRs, SNPs and Indels^122^.

As a model work, mapping of transcripts over two well-known root drought trait QTLs of wheat present on chromosome 3B (*Xbarc268- Xbarc075* and *Xbarc102-Xbarc268*) revealed a total of 18 genes. Among these genes, 9 each were up and down- regulated, respectively. BLAST analysis (blastx) revealed 8 transcripts with known function related to drought response and 10 novel wheat specific novel proteins which are yet to be functionally categorized (Supplementary Tλable S9). Interestingly, maximum SNPs were present in two transcripts only, viz., gene Ubiquitin-protein ligase SINAT5 (c130535_g1_i8) and a hypothetical gene (c142983_g2_i1). Former gene is well known for regulation of lateral root formation along with down regulation of auxin signal^123^. This clearly dissects QTL region into QTN but such findings need further association studies and validation before using them as marker. Such high resolution of QTL into QTN can be much more effective in breeding programs^16^.

In the present investigation, we have successfully demonstrated such applications by taking the model example of chromosome 3B where maximum drought responsive QTL of wheat seedling stage are reported which controls trait like root to shoot fresh weight under osmotic stress^76^. Our web genomic resources can be used to cover all chromosomes by similar approach to map all reported drought root specific QTL regions to discover more putative candidate genes related to this trait. These genes can be further used for targeted SNP discovery in large varietal population for marker association studies. Such use of transcriptome data of contrasting varieties in dissection and validation of QTL along with discovery of candidate genes has been reported in other cereal crop like barley also^124^.

### SSR Markers discovery

SSRs were mined from *de novo* transcriptome assembly and a total of 28807 SSR markers were found, out of which 1059 were in compound formation. We found 2808 transcripts containing more than 1 SSR markers. A total of 9296, 6348, 12143, 913, 79 and 28 SSR markers were obtained in mononucleotides, dinucleotides, trinucleotides, tetranucleotides, pentanucleotides and hexanucleotides, respectively. These results revealed that maximum number markers were mined from trinucleotides, followed by mononucleotides and dinucleotides. Using PRIMER3 tool, three sets of primers were designed from 21571 markers for future perspective. Moreover, we also identified markers and primers from 4 sets of differential expressed genes viz. TC:TD, SD:TD, SC:SD and SC:TC (Table 6, Supplementary Table S10).

**Table 6:** Markers obtained from four sets of differential expressed genes.

	TC:TD	SD:TD	SC:SD	SC:TC
Total number of sequences examined	17798	8103	9910	9328
Total number of identified SSRs	1179	1123	751	1001
Number of SSR containing sequences	1016	968	637	861
Number of sequences containing more than 1 SSR	131	132	91	120
Number of SSRs present in compound formation	61	48	36	49
Mono	439	412	243	365
Di	236	273	178	233
Tri	469	410	302	367
Tetra	28	23	21	28
Penta	5	4	4	4
Hexa	2	1	3	4

### Validation of SSR markers

**Figure 4:**
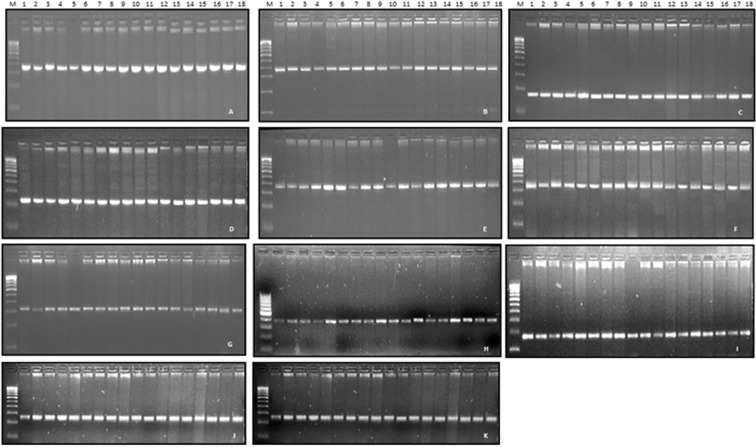
Validation of identified genic SSRs localized on differentially expressed transcripts in wheat genotypes: (**A**) pwtssr 3, (**B**) pwtssr 5, (**C**) pwtssr 6, (**D**) pwtssr 9, (**E**) pwtssr 10, (**F**) pwtssr 12, (**G**) pwtssr 14, (**H**) pwtssr 16, (**I**) pwtssr 17, (**J**) pwtssr 19, (**K**) pwtssr 20; M is 100 bp ladder used as a standard marker.

A panel of eighteen highly diverse wheat genotypes was selected from the mini-core set developed for the drought tolerance for validation of SSR loci (Supplementary Table S11). Out of the 15 loci selected for designing the primer, a total of 11 SSR loci were successful in generating PCR products. Since the resolution power of gel used is limited (>5 bp), thus limited polymorphism was seen in few loci (Fig. 4). Genic region SSR markers can be used in crop improvement program. Such DEG based SSR discovery has been reported in wheat against abiotic stress cold tolerance^125^ and dormancy^126^. Similar use of genic region markers are also reported in other crops like switchgrass for rust resistance^127^ and *Brassica*^128^.

### Variants discovery

**Figure 5:**
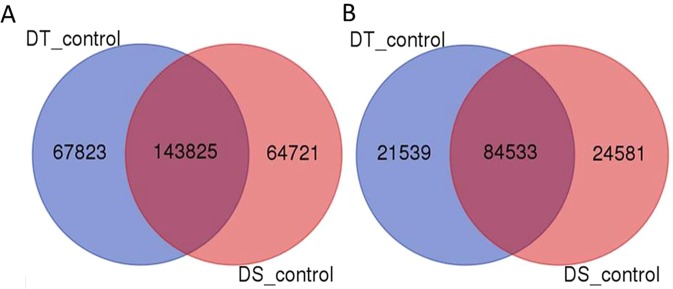
Venn diagram of common and unique variants obtained from (**A**) *de novo* transcriptome assembly and (**B**) wheat reference genome.

All the DEGs were further subjected to SNP discovery using both the references, namely, our constructed wheat *de novo* transcriptome assembly and wheat genome release version 31. Analysis revealed that 143825 SNPs and InDels were common in control samples, whereas 67823 and 64721 variants were found unique in control samples NI5439 (drought tolerant) and WL711 (drought susceptible), respectively. Further from reference genome approach, a total of 21539 and 24581 variants were obtained which were unique to genotype NI5439 and WL711, respectively and 84533 common to both (Fig. 5). Relative distribution of SNPs and InDels were obtained by both the approaches available at (http://webtom.cabgrid.res.in/wdrotdb/). All these variants can be used as genomic resource for future association studies.

Visualization of chromosome wise SNP distribution over 21 chromosomes of wheat revealed maximum number of SNPs on chromosome 3B. This is obviously expected due to its largest size in the genome assembly (version 31) used. The other potential reason for this could be use of drought tolerant contrasting genotype to generate transcriptomic data. When selection increases frequency of favoured alleles in varietal population then due to genetic hitchhiking, neighbouring link having variations gets reduced by selection sweep^129^. Since SNP alleles are fixed in well selected contrasting crop varieties due to such selection sweep^130^, thus contrasting varieties are expected to yield more SNPs.

**Figure 6:**
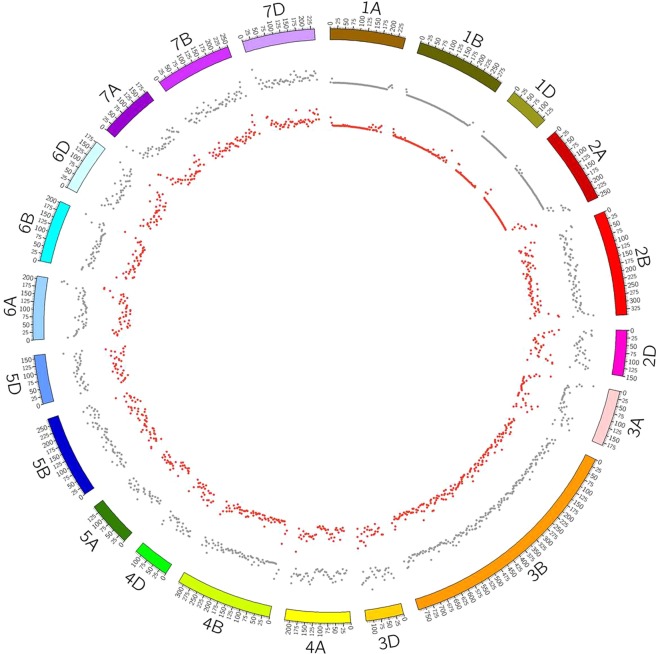
Chromosome wise SNP distribution over 21 chromosomes of wheat by circular plot. Grey dots (drought tolerant) and red dots (drought susceptible).

Minimum number of SNPs were present on the smallest chromosome 4D. Relative distribution of SNPs over 21 chromosomes of wheat genome revealed that out of 83850 SNP discovered in this study hardly one third (31657) of them (37.75%) only gets mapped (Fig. 6). It is interesting to note that in genic region SNP discovery in wheat, transcriptome assembly approach is advantageous as it discovers much higher number (more than two folds) as reported in present study. Even whole genome re-sequencing of these two varieties followed by reference based SNP discovery approach would have missed these additional SNPs. Highest and lowest SNPs were found on chromosome 3B and 1D, respectively. This is due to the size of respective chromosome as 3B is largest and 1D is smallest in the reference assembly used for SNP discovery. SNP discovered in wheat transcriptome can be used for linkage analysis for genetic map construction^131^. Genic region SNP marker has also been used for cost effective, rapid genotyping by derived CAPS (dCAPS) approach to identify crop germplasm like radish^132^.

### Web genomic resources

**Figure 7:**
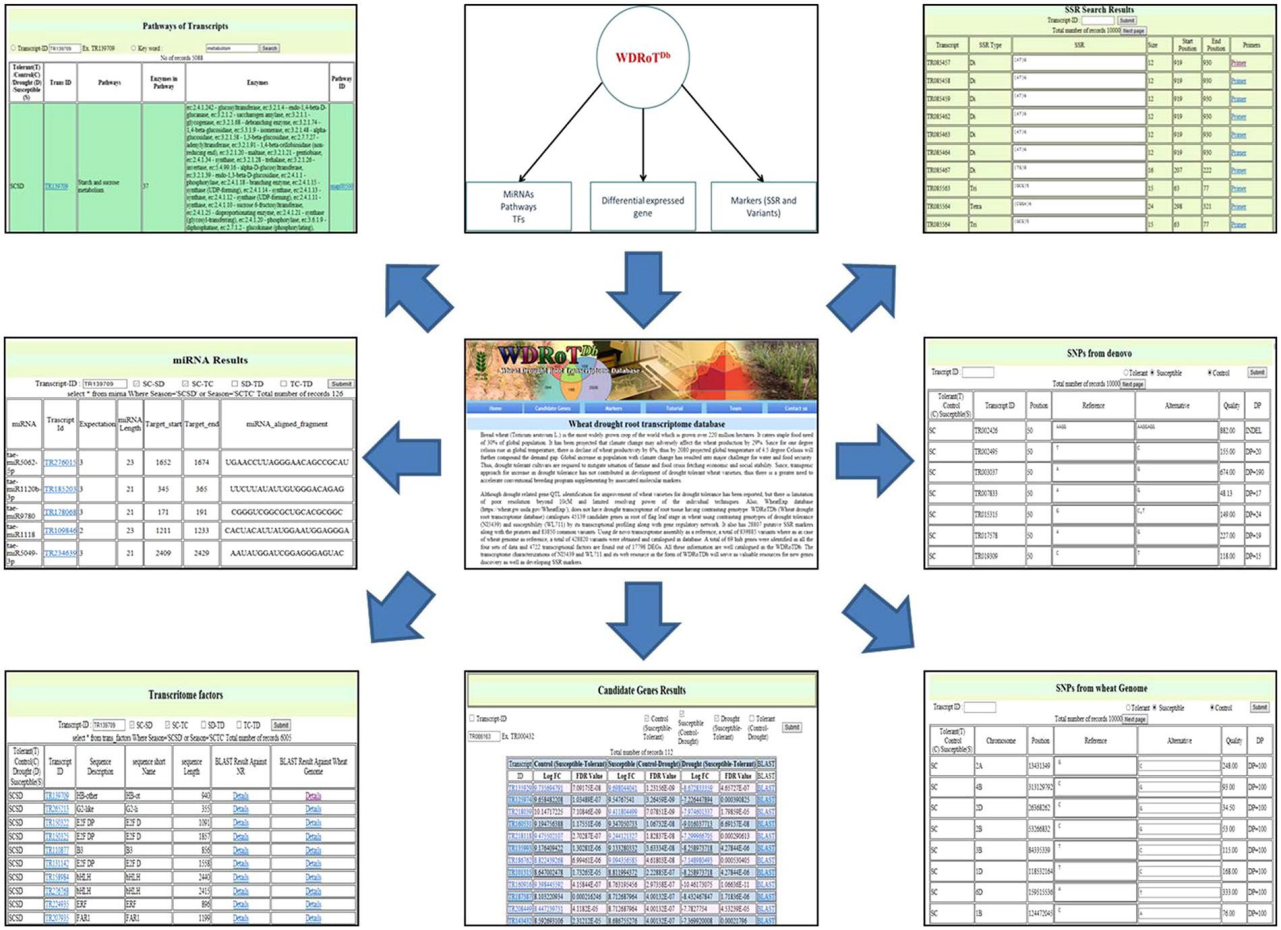
Web interface of WDRoTDb showing search option for candidate genes, variants, transcripts expression profile and miRNA targets.

WDRoTDb is equipped with six tables, viz., Home, Candidate genes (having DEGs, miRNAs, TF and KEGG pathway), markers (SSRs and SNPs), Tutorial, Team and Contact). Users can have the details of DEGs, miRNA targets, associated transcription factors and details of the KEGG pathway from the “Candidate genes” tab. Provision of *in silico* mining of chromosome specific SSR markers, including motif type, kind, copy number, base pair, percentage GC content along with physical location (start and end) of microsatellite markers. Figure 7 shows the interface for the usage of this tool.

## Conclusion

We report the molecular mechanism of wheat root drought responsiveness by irrigation withdrawal method using contrasting varieties at Zadok’s scale (Z24- Z37) scale which is critical for drought resilience. Study reveals a total of 45139 DEGs, 13820 TF, 288 miRNAs, 640 pathways, 435829 putative markers (28807 SSRs, 276369 and 130653) variants from *de novo* and reference based, respectively. Study also reports 67823 and 64721 contrasting variety specific variants for drought tolerant and drought susceptible varieties, respectively which are relevant for future association studies. As a model work, we demonstrate that QTL region can be further dissected by RNA Seq data to understand its role in terms of DEG of specific genes harbouring in QTL regions along with its structural variants in terms of SNPs and InDels. GRNs constructed revealed role of key candidate genes responding to drought. We also demonstrate the putative use of genomic resources by wet lab validation of 11 SSR loci in 18 diverse genotypic set of wheat.

## Supplementary information

Supplementary Information

Supplementary Table S1

Supplementary Table S2

Supplementary Table S3

Supplementary Table S4

Supplementary Table S5

Supplementary Table S6

Supplementary Table S7

Supplementary Table S8

Supplementary Table S9

Supplementary Table S10

Supplementary Table S11

## Data Availability

The RNA-Seq dataset used in this study are available in the NCBI repository with following accessions and is kept at hold till the publication. These would be made public after publication. BioProject: PRJNA432496 BioSamples: SAMN08450194, SAMN08450195, SAMN08450196, SAMN08450197 All the supplementary data are available for download at http://webtom.cabgrid.res.in/wdrotdb/.
