# Supplementary material for: RNAseq analysis reveals drought-responsive molecular pathways with candidate genes and putative molecular markers in root tissue of wheat
Source: Sci Rep. 2019 Sep 26;9:13917. doi: 10.1038/s41598-019-49915-2 (PMC6763491; doi:10.1038/s41598-019-49915-2)
Supplement: Supplementary file 1 — Supplementary Information [file 41598_2019_49915_MOESM1_ESM.docx]

**RNAseq analysis reveals drought-responsive molecular pathways with candidate genes and putative molecular markers in root tissue of wheat**

Mir Asif Iquebal^1,#^, Pradeep Sharma^2,#^, Rahul Singh Jasrotia^1^, Sarika Jaiswal^1^, Amandeep Kaur^2^, Monika Siroha^2^, UB Angadi^1^, Sonia Sheoran^2^, Rajender Singh^2^, GP Singh^2^, Anil Rai^1^, Ratan Tiwari^2,*^, Dinesh Kumar^1,*^

^1^Centre for Agricultural Bioinformatics, ICAR-Indian Agricultural Statistics Research Institute, Library Avenue, PUSA, New Delhi-110012, INDIA

^2^ICAR-Indian Institute of Wheat and Barley Research, Karnal, Haryana-132001, INDIA

**List of Supplementary Tables**

**Supplementary Table S1:** Root traits of contrasting varieties grown in PVC pipes under well-watered and drought stress condition

**Supplementary Table S2:** List of significant differentially expressed genes in wheat drought transcriptome

**Supplementary Table S3:** Pathways identified in wheat root transcriptome associated with drought

**Supplementary Table S4:** List of 101 candidate genes common to all four sets of wheat drought transcriptome

**Supplementary Table S5:** qRT-PCR analysis of randomly selected wheat drought transcripts

**Supplementary Table S6:** Gene Network Analysis: Subnetwork of important Hub genes in wheat drought transcriptome

**Supplementary Table S7:** Blast hits of identified transcription factors in wheat root transcriptome associated with drought

**Supplementary Table S8:** Blast results of identified miRNA with their targets in wheat root transcriptome associated with drought

**Supplementary Table S9**: List of 18 genes identified after mapping of transcripts over two root drought trait QTLs (Xbarc268- Xbarc075 and Xbarc102-Xbarc268) of wheat on chromosome 3B

**Supplementary Table S10:** List of SSR markers mined from de novo transcriptome assembly of wheat

**Supplementary Table S11:** List of genotypes used for SSRs validation
