# Supplementary material for: RNAseq analysis reveals drought-responsive molecular pathways with candidate genes and putative molecular markers in root tissue of wheat
Source: Sci Rep. 2019 Sep 26;9:13917. doi: 10.1038/s41598-019-49915-2 (PMC6763491; doi:10.1038/s41598-019-49915-2)
Supplement: Supplementary file 2 — Supplementary Table S1 [file 41598_2019_49915_MOESM2_ESM.docx]

**RNAseq analysis reveals drought-responsive molecular pathways with candidate genes and putative molecular markers in root tissue of wheat**

Mir Asif Iquebal^1,#^, Pradeep Sharma^2,#^, Rahul Singh Jasrotia^1^, Sarika Jaiswal^1^, Amandeep Kaur^2^, Monika Siroha^2^, UB Angadi^1^, Sonia Sheoran^2^, Rajender Singh^2^, GP Singh^2^, Anil Rai^1^, Ratan Tiwari^2,*^, Dinesh Kumar^1,*^

^1^Centre for Agricultural Bioinformatics, ICAR-Indian Agricultural Statistics Research Institute, Library Avenue, PUSA, New Delhi-110012, INDIA

^2^ICAR-Indian Institute of Wheat and Barley Research, Karnal, Haryana-132001, INDIA

**Supplementary Table S1.** Root traits of contrasting varieties grown in PVC pipes under well-watered and drought stress condition

|  |  | NI5439 (DT) |  |  | WL711 (DS) |  |  |
| --- | --- | --- | --- | --- | --- | --- | --- |
|  |  | DT | IR | Sig diff | DT | IR | Sig diff |
| Root length (cm) | 0-30 | 2392.91±324.33 | 2191.83±114.48 | ns | 2152.20±15.01 | 1821.66±23.99 | * |
|  | 30-60 | 814.00±10.47 | 531.83±31.69 | * | 701.83±49.43 | 1076.67±149.44 | ns |
|  | 60-90 | 799.51±112.08 | 675.27±99.93 | ns | 604.20±10.97 | 1006.60±89.65 | ns |
|  | 90-120 | 734.23±77.74 | 1086.76±123.35 | ns | 504.84±14.46 | 809.91±3.64 | * |
| Total Surface Area (cm^2^) | 0-30 | 156.05±20.95 | 132.37±0.64 | ns | 147.83±4.31 | 108.70±0.38 | ns |
|  | 30-60 | 57.49±4.57 | 34.93±3.29 | * | 49.09±3.75 | 71.24±10.70 | ns |
|  | 60-90 | 49.65±6.43 | 45.80±5.94 | ns | 42.54±1.70 | 66.94±5.30 | ns |
|  | 90-120 | 47.05±4.92 | 69.91±7.77 | ns | 37.26±3.77 | 54.47±3.34 | ns |
| Average diameter (mm) | 0-30 | 8.72±3.92 | 5.75±0.09 | ns | 11.31±1.09 | 5.37±1.41 | ns |
|  | 30-60 | 3.22±0.17 | 1.12±0.06 | ns | 1.76±0.03 | 3.25±0.02 | * |
|  | 60-90 | 4.43±0.58 | 3.89±0.89 | ns | 2.90±0.38 | 2.96±1.33 | ns |
|  | 90-120 | 3.80±0.82 | 3.41±0.84 | ns | 2.58±0.18 | 7.68±0.49 | ns |
| Length/volume (cm/m^3^) | 0-30 | 4938.74±1104.43 | 3949.70±2122.65 | ns | 6286.92±2.66 | 2071.11±982.50 | ns |
|  | 30-60 | 5393.12±315.01 | 937.32±38.93 | * | 5257.56±277.35 | 7445.68±913.99 | ns |
|  | 60-90 | 4824.07±867.74 | 3725.88±136.96 | ns | 4582.06±236.34 | 6393.11±197.26 | ns |
|  | 90-120 | 4565.64±99.42 | 5722.59±3080.52 | ns | 3795.8±371.41 | 2988.95±152.59 | ns |

**RL: Root length (cm); TSA: Total Surface Area (cm^2^); AD: Average diameter (mm); L/V: Length/volume (cm/m^3^)**

**DT: Drought, IR: Irrigation; ns= non-significant**

* Symbol of up and down arrow represents increase and decrease in value at 5% level of significance
