# Supplementary material for: RNAseq analysis reveals drought-responsive molecular pathways with candidate genes and putative molecular markers in root tissue of wheat
Source: Sci Rep. 2019 Sep 26;9:13917. doi: 10.1038/s41598-019-49915-2 (PMC6763491; doi:10.1038/s41598-019-49915-2)
Supplement: Supplementary file 12 — Supplementary Table S11 [file 41598_2019_49915_MOESM12_ESM.docx]

**RNAseq analysis reveals drought-responsive molecular pathways with candidate genes and putative molecular markers in root tissue of wheat**

Mir Asif Iquebal^1,#^, Pradeep Sharma^2,#^, Rahul Singh Jasrotia^1^, Sarika Jaiswal^1^, Amandeep Kaur^2^, Monika Siroha^2^, UB Angadi^1^, Sonia Sheoran^2^, Rajender Singh^2^, GP Singh^2^, Anil Rai^1^, Ratan Tiwari^2,*^, Dinesh Kumar^1,*^

^1^Centre for Agricultural Bioinformatics, ICAR-Indian Agricultural Statistics Research Institute, Library Avenue, PUSA, New Delhi-110012, INDIA

^2^ICAR-Indian Institute of Wheat and Barley Research, Karnal, Haryana-132001, INDIA

**Supplementary Table S11: List of genotypes used for SSRs validation**

| **Sr No.** | **Genotype** | **Pedigree** | **Production condition** | **Reference** |
| --- | --- | --- | --- | --- |
| 1 | DWR 39 | LR64/2*SN64//S308 | Timely Sown, Irrigated | Kundu et al. 2006 |
| 2 | MACS 2694 | Mexicali/Raj 1555//MACS 2130 | Timely Sown, Irrigated | Kundu et al. 2006 |
| 3 | HD 2643 | VEE'S'/HD2407/HD2329 | Late Sown, Irrigated | Kundu et al. 2006 |
| 4 | K 8962 | K7401/HD2160 | Timely Sown, Rainfed drought tolerant | Kundu et al. 2006; Kumar et al. 2015 |
| 5 | D 134 | RS 31/ WIS 245’S’ | Timely Sown, Rainfed | Kundu et al. 2006 |
| 6 | RAJ 4356 |  | Timely Sown, Rainfed | Kundu et al. 2006 |
| 7 | PBW 175 | HD 2160/ WG 1025 | Timely Sown, Rainfed, drought tolerant | Kundu et al. 2006; Rakhra et al. 2017 |
| 8 | HI 1500 | HW 2002*2/STREMPALLI/PNC 5 | Timely Sown, Rainfed, drought tolerant | Kundu et al. 2006; Rai et al. 2017 |
| 9 | WL 711 | NP/TOB’S’/3/8156//KAL/BB | Timely Sown, Irrigated; drought susceptible | Kundu et al. 2006; Kadam et al 2012 |
| 10 | WH 542 | JUPATECO/BLUE/JAY//URES | Timely Sown, Irrigated, drought susceptible | Kundu et al. 2006; Munjal and Dhanda, 2006 |
| 11 | UP 2887 | CPAN4022/UP2382)/N/II58.57//PRL/3/AHN/4/KAUZ’5’/KAUZ | Timely sown, Irrigated | Gupta et al 2017 |
| 12 | K 9351 | K 72/K 8077//K 72 | Timely Sown, Rainfed | Kundu et al. 2006 |
| 13 | UP 2338 | UP 36/VL 42l//UP 262 | Timely Sown /Late sown Irrigated | Kundu et al. 2006 |
| 14 | DWR 162 | KVZ/BUHO//KAL/BB | Timely Sown, Irrigated | Kundu et al. 2006 |
| 15 | WH 291 | HD1925/3/N10B/K68//BB | Late sown, Irrigated | Kundu et al. 2006 |
| 16 | HI 385 | HYB 633//Gaza(DR)/KPD25 | Timely Sown, Rainfed, drought tolerant | Kundu et al. 2006 Pant et al 2018 |
| 17 | UP 2554 | SM4-HSN 24E/CPAN |  | Gupta et al 2017 |
| 18 | HUW 510 | HD 2278 / HUW 234 // DL 230 - 16 | Timely Sown, Irrigated, drought susceptible | Kundu et al. 2006  Ramya et al 2016 |

**Reference:**

Kundu S, Jag Sheoran, Mishra B, Gupta R.K. . 2006. Indian wheat varieties at a glance. Directorate of Wheat Research, Karnal-132001, India. Research Bulletin No.21, P: 447.

Arun Gupta, Charan Singh,Vineet Kumar, Sushila Kundu, Vinod Tiwari and G.P Singh.2017. Indian wheat varieties at a glance, Vol2 (Varieties released between 2006-2017. ICAR-IIWBR, Karnal-132001, India: 156pp

[Kumar](https://www.researchgate.net/scientific-contributions/2117648192_A_Kumar?_sg=fTDGzmEP6t81ghNfTWJHl1Cq9oRhkT9GJC_xrOpKZENG9C9UcU1GMujhXRnSePdxnmESBh8.ThzrR2J9BhBLddK4hOxtblb6vitwSL3ZRKdHgvtA7FtxVaZmd4nTlAs5ejsI_ZJC5TsUHNOMAq9ZwPqDOP-ujQ) A.,  [Singh](https://www.researchgate.net/scientific-contributions/2054706442_NB_Singh?_sg=fTDGzmEP6t81ghNfTWJHl1Cq9oRhkT9GJC_xrOpKZENG9C9UcU1GMujhXRnSePdxnmESBh8.ThzrR2J9BhBLddK4hOxtblb6vitwSL3ZRKdHgvtA7FtxVaZmd4nTlAs5ejsI_ZJC5TsUHNOMAq9ZwPqDOP-ujQ) N.B., [Kumar](https://www.researchgate.net/scientific-contributions/2082939435_K_Kumar?_sg=fTDGzmEP6t81ghNfTWJHl1Cq9oRhkT9GJC_xrOpKZENG9C9UcU1GMujhXRnSePdxnmESBh8.ThzrR2J9BhBLddK4hOxtblb6vitwSL3ZRKdHgvtA7FtxVaZmd4nTlAs5ejsI_ZJC5TsUHNOMAq9ZwPqDOP-ujQ) L.K. 2015. Variability in biochemical traits associated with drought and heat tolerance in wheat genotypes under different irrigation level. *Indian Journal of Agricultural Biochemistry* 28(1):48-51

Rai, N., Amasiddha, B., Jain, N., Singh, G. P., Singh, P. K., Chand, S., and Prabhu, K. V., Validation of SSR Markers Linked with Drought and Heat Tolerant QTLs in Bread Wheat (Triticum aestivum L. em.Thell.), *Int. J. Pure App. Biosci*. 5(5): 700-705 (2017). doi: [http://dx.doi.org/10.18782/2320- 7051.5611](http://dx.doi.org/10.18782/2320-%207051.5611)

Ramya P., Singh GP, Jain N, Singh PK, Pandey MK, Sharma K, et al. (2016) Effect of Recurrent Selection on Drought Tolerance and Related Morpho-Physiological Traits in Bread Wheat. *PLoS ONE* 11(6): e0156869. <https://doi.org/10.1371/journal.pone.0156869>

Pant U and Khanna V.K.. 2018. Diversity Analysis and Hybridity Test of Drought Responsive Wheat Cultivars and Their F1’s on the Basis of SDS-PAGE Profiles. *Int.J.Curr.Microbiol.App.Sci*. 7(06): 3319-3335. doi: <https://doi.org/10.20546/ijcmas.2018.706.389>

[Rakhra G](https://www.ncbi.nlm.nih.gov/pubmed/?term=Rakhra%20G%5BAuthor%5D&cauthor=true&cauthor_uid=28033539), [Kaur T](https://www.ncbi.nlm.nih.gov/pubmed/?term=Kaur%20T%5BAuthor%5D&cauthor=true&cauthor_uid=28033539), [Vyas D](https://www.ncbi.nlm.nih.gov/pubmed/?term=Vyas%20D%5BAuthor%5D&cauthor=true&cauthor_uid=28033539), [Sharma AD](https://www.ncbi.nlm.nih.gov/pubmed/?term=Sharma%20AD%5BAuthor%5D&cauthor=true&cauthor_uid=28033539), [Singh J](https://www.ncbi.nlm.nih.gov/pubmed/?term=Singh%20J%5BAuthor%5D&cauthor=true&cauthor_uid=28033539), [Ram G](https://www.ncbi.nlm.nih.gov/pubmed/?term=Ram%20G%5BAuthor%5D&cauthor=true&cauthor_uid=28033539) . 2017. Molecular cloning, characterization, heterologous expression and in-silico analysis of disordered boiling soluble stress-responsive wBsSRP protein from drought tolerant wheat cv.PBW 175. [*Plant Physiol Biochem.*](https://www.ncbi.nlm.nih.gov/pubmed/28033539) 2017 Mar;112:29-44. doi: 10.1016/j.plaphy.2016.12.017.

[Kadam S](https://www.ncbi.nlm.nih.gov/pubmed/?term=Kadam%20S%5BAuthor%5D&cauthor=true&cauthor_uid=22476619), [Singh K](https://www.ncbi.nlm.nih.gov/pubmed/?term=Singh%20K%5BAuthor%5D&cauthor=true&cauthor_uid=22476619), [Shukla S](https://www.ncbi.nlm.nih.gov/pubmed/?term=Shukla%20S%5BAuthor%5D&cauthor=true&cauthor_uid=22476619), [Goel S](https://www.ncbi.nlm.nih.gov/pubmed/?term=Goel%20S%5BAuthor%5D&cauthor=true&cauthor_uid=22476619), [Vikram P](https://www.ncbi.nlm.nih.gov/pubmed/?term=Vikram%20P%5BAuthor%5D&cauthor=true&cauthor_uid=22476619), [Pawar V](https://www.ncbi.nlm.nih.gov/pubmed/?term=Pawar%20V%5BAuthor%5D&cauthor=true&cauthor_uid=22476619), [Gaikwad K](https://www.ncbi.nlm.nih.gov/pubmed/?term=Gaikwad%20K%5BAuthor%5D&cauthor=true&cauthor_uid=22476619), [Khanna-Chopra R](https://www.ncbi.nlm.nih.gov/pubmed/?term=Khanna-Chopra%20R%5BAuthor%5D&cauthor=true&cauthor_uid=22476619), [Singh N](https://www.ncbi.nlm.nih.gov/pubmed/?term=Singh%20N%5BAuthor%5D&cauthor=true&cauthor_uid=22476619).2012. Genomic associations for drought tolerance on the short arm of wheat chromosome 4B. [*Funct Integr Genomics.*](https://www.ncbi.nlm.nih.gov/pubmed/22476619) 2012 Aug;12(3):447-64. doi: 10.1007/s10142-012-0276-1.

Munjal R., Dhanda S. S., 2016. Assessment of drought resistance in Indian wheat cultivars for morpho-physiological traits. *Ekin J.* 2(1):74-81.
